# Health diagnosis associated with COVID-19 death in the United States: A retrospective cohort study using electronic health records

**DOI:** 10.1371/journal.pone.0319585

**Published:** 2025-03-31

**Authors:** Mariam Joseph, Qiwei Li, Sunyoung Shin

**Affiliations:** 1 Department of Statistics, University of Michigan, Ann Arbor, Michigan, United States of America; 2 Department of Mathematical Sciences, University of Texas at Dallas, Richardson, Texas, United States of America; 3 Department of Mathematics, Pohang University of Science and Technology, Pohang, Gyeongbuk, South Korea; Chipata Central Hospital, ZAMBIA

## Abstract

**Background:**

The United States has experienced high surge in COVID-19 cases since the dawn of 2020. Identifying the types of diagnoses that pose a risk in leading COVID-19 death casualties will enable our community to obtain a better perspective in identifying the most vulnerable populations and enable these populations to implement better precautionary measures.

**Objective:**

To identify demographic factors and health diagnosis codes that pose a high or a low risk to COVID-19 death from individual health record data sourced from the United States.

**Methods:**

We used logistic regression models to analyze the top 500 health diagnosis codes and demographics that have been identified as being associated with COVID-19 death.

**Results:**

Among 223,286 patients tested positive at least once, 218,831 (98%) patients were alive and 4,455 (2%) patients died during the duration of the study period. Through our logistic regression analysis, four demographic characteristics of patients; age, gender, race and region, were deemed to be associated with COVID-19 mortality. Patients from the West region of the United States: Alaska, Arizona, California, Colorado, Hawaii, Idaho, Montana, Nevada, New Mexico, Oregon, Utah, Washington, and Wyoming had the highest odds ratio of COVID-19 mortality across the United States. In terms of diagnoses, *Complications mainly related to pregnancy* (Adjusted Odds Ratio, OR:2.95; 95% Confidence Interval, CI:1.4 - 6.23*)* hold the highest odds ratio in influencing COVID-19 death followed by *Other diseases of the respiratory system* (OR:2.0; CI:1.84 – 2.18)*, Renal failure* (OR:1.76; CI:1.61 – 1.93)*, Influenza and pneumonia* (OR:1.53; CI:1.41 – 1.67)*, Other bacterial diseases* (OR:1.45; CI:1.31 – 1.61)*, Coagulation defects, purpura and other hemorrhagic conditions*(OR:1.37; CI:1.22 – 1.54)*, Injuries to the head* (OR:1.27; CI:1.1 - 1.46), *Mood [affective] disorders* (OR:1.24; CI:1.12 – 1.36*), Aplastic and other anemias* (OR:1.22; CI:1.12 – 1.34)*, Chronic obstructive pulmonary disease and allied conditions* (OR:1.18; CI:1.06 – 1.32)*, Other forms of heart disease* (OR:1.18; CI:1.09 – 1.28)*, Infections of the skin and subcutaneous tissue* (OR: 1.15; CI:1.04 – 1.27), *Diabetes mellitus* (OR:1.14; CI:1.03 – 1.26)*, and Other diseases of the urinary system* (OR:1.12; CI:1.03 – 1.21).

**Conclusion:**

We found demographic factors and medical conditions, including some novel ones which are associated with COVID-19 death. These findings can be used for clinical and public awareness and for future research purposes.

## Introduction

The COVID-19 (Coronavirus Diseases 2019) pandemic has been substantially impacting most individuals in the world since it broke out in Wuhan, China in December 2019. The COVID-19 caused by acute respiratory syndrome coronavirus 2 (SARS-CoV-2) is highly contagious and can lead to death. The U.S. Centers for Disease Control and Prevention (U.S. CDC) declared the COVID-19 pandemic the third most popular leading cause of death in the United States by the end of 2020 [1]. The case-fatality ratio, which is the number of deaths divided by the number of confirmed cases, in the U.S. is 1.1% [2]. The severity of the COVID-19 varies among individuals. Many COVID-19 patients have mild illness, and some patients are asymptomatic; however, others face serious consequences such as hospitalization, admission to intensive care or death [3]. Adults over the age of 65 with pre-existing medical conditions such as obesity, diabetes, asthma, hypertension, cardiovascular diseases, or chronic lung diseases have been found to be at higher risk of severe COVID-19 outcomes [4–6].

Comprehensive systematic review and meta-analysis have been conducted for quantitative evaluation of the impact of such pre-existing medical conditions on the COVID-19 mortality. During the rise of the pandemic, COVID-19 hospitalizations/ICU admission data obtained from multiple studies were synthesized and analyzed through meta-analysis with random-effects, which identified that hypertension, coronary heart disease, diabetes, lymphocytopenia and D-dimer are factors associated with increased mortality during hospitalization [7–9]. With an increase in amount of COVID-19 data accumulated, many systematic reviews on multiple studies published have identified underlying health conditions as prognostic factors for the COVID-19 mortality [3,10–12]. However, different data sources that have inconsistent definitions of outcomes, selection criteria, reporting, etc. make the interpretations of the combined results from the multiple studies difficult [3,13].

COVID-19 electronic health records (EHR) data collected from large multicenter cohort studies contain crucial pieces of information to understand underlying health factors determining COVID-19 hospitalization and mortality. The large volume of CERNER EHR database has been widely used to understand the risk of COVID-19 among patients with specific conditions such as patients with sickle cell disease, pregnant women, and patients with type 1 diabetes [14–16]. A U.S. healthcare company OPTUM initiated individual-level COVID-19 EHR data collection in February 2020, near the beginning of the pandemic, harnessing the power of comprehensive medical networks. A full access to the OPTUM database has been granted to UTHealth Systems and UTHealth School of Biomedical Informatics (SBMI) Data Service. It has made biweekly updates. The OPTUM EHR database has been exploited by researchers who conduct in-depth investigations on patients with COVID-19 [17–21]. Characteristics of adults hospitalized with COVID-19 were examined along with their disease progression and outcomes, and their changes were explored over time [18,20,21]. [17] studied patients’ clinical conditions after COVID-19 diagnosis or hospital discharge and recognized the significance of their adverse long-term outcomes. The EHR data have provided evidence that patients with psychiatric disorders are at higher risk of COVID-19 infection and mortality [19,22].

The rich OPTUM EHR data source includes full demographics and baseline characteristics of COVID-19 deaths such as comorbidities and medication use collected during the enrollment period [23]. Statistical models containing the demographics and the baseline characteristics for COVID-19 outcomes were used for predicting prognosis of hospitalized adults with COVID-19 and understanding racial and ethnic disparity in clinical outcomes among the patients [18,24]. Acute respiratory failure, pneumonia, sepsis, coagulation defects, arrhythmia, and myocardial infarction are found to be the recent diagnoses predictive of COVID-19 deaths during hospitalization [25].

The objective of this paper is to assess the effect of pre-existing medical conditions that determine the mortality in COVID-19 patients. We leverage full access to the individual-level medical diagnosis records for up to 10 years with standard codes in common use in the OPTUM EHR database. [26] considered all subjects tested for COVID-19 in the OPTUM medical networks between February, 2020 and August, 2020 regardless of their COVID-19 infection. In this paper, our focus is COVID-19 patients since we aim to improve accuracy in evaluating associations between the pre-existing medical conditions and the mortality among COVID-19 cases. The study subjects are divided into alive and died groups. The associations between COVID-19 mortality and the demographic factors such as age, regions, and race are interrogated. Logistic models adjusted for the demographics are used to identify COVID-19 patients’ pre-existing diagnoses that increase risk of deaths. Identifying populations with underlying conditions that are associated with the COVID-19 mortality would help healthcare providers to provide better medical interventions to COVID-19 patients.

## Methods

### Study design

OPTUM, an American health care provider, acquired COVID-19 EHR data from various medical care provider organizations which include hospitals and clinics across the United States since February 2020. The observational COVID-19 EHR dataset consists of medical and healthcare utilization data from outpatient, inpatient and ambulatory medical records, medical practice management systems, and several other internal systems. An independent statistical expert has certified the data to be de-identified based on the HIPAA statistical de-identification rules and OPTUM customer data use agreements. The study protocol was exempt from review in a written form by the Institutional Review Board (IRB) at University of Texas at Dallas. The IRB exemption was received since any information containing personally identifying information is not collected nor obtained, and the identity and privacy of study participants are protected by reviewing the de-identified data in a private setting during each phase of research. Our study followed the Strengthening the Reporting of Observational Studies in Epidemiology (STROBE) reporting guideline.

### Study population and observation period

The COVID-19 dataset used for this study was collected from February 2020 until January 2021 and contains 2,627,679 individuals (COVID-19 positive and negative). However, for this study, we exclusively analyzed COVID-19 positive patients, removing all COVID-19 negative individuals from the dataset. The dataset includes 223,286 COVID-19 positive individuals who meet at least one of the following criteria: (i) have taken a laboratory test for COVID-19 or a COVID-19 antibody test, (ii) have been associated with a procedure code for COVID-19 test, and (iii) possess COVID-19 diagnosis code or diagnosis code to a similar condition in their respective medical records. For these individuals, the EHR was included up to 10 years prior to their COVID-19 diagnosis, where available. Patient deaths are recorded with their month and year of death. Additionally, history of patient visits, procedures, laboratory diagnostics, and observations are recorded to analyze treatments.

### Data preprocessing

We conducted the COVID-19 data preprocessing and analysis from March 2021 until December 2022. Unix shell scripts were written to obtain COVID-19 patient information from the initial OPTUM COVID-19 EHR dataset. Specifically, we used the information about lab and test results to acquire the patients that were COVID-19 positive along with their ID, COVID-19 test order date, and test collected date. We acquired the following demographic variables: age, gender, race, ethnicity, and region. The variable race has three levels: African American, Asian, and Caucasian. The variable ethnicity is about whether an individual is either Hispanic, or Not Hispanic. The variable gender takes Female or Male. Region is primarily based on the categorization of states by the United States Census Bureau which are Northeast, Midwest, West, and South [27].

As shown in S1 Fig, we excluded 300,971 patients out of 2,627,679 (11.45%) who identified unknown in at least one of the demographic categories. Patients’ death records, identified as YYYYMM format, were also acquired. Additionally, the information about the patients’ visit was processed for obtaining their last visit date that enabled us to obtain a calculated estimate of the patients’ age who survived COVID-19. Lastly, we obtained all patients’ pre-existing conditions from their diagnosis history before May 5, 2020. This cut-off date was chosen to ensure feature engineering of all patients’ diagnosis history files identified in OPTUM Data. We obtained 223,286 individuals who tested positive for COVID-19 at least once from the sample of 2,326,708 individuals who took COVID-19 tests.

### Diagnostic measures

In this study, we identified the top 500 diagnosis codes most frequently used among COVID-19 patients within the study period. These codes were then standardized and aligned with the International Classification of Diseases (ICD) versions 9 and 10. This preprocessing step was essential to ensure consistency in diagnosis categorization across different coding systems. To further refine our analysis, we consolidated these codes into 117 unique ‘pre-existing diagnosis codes.’ This consolidation involved merging related ICD 9 and 10 diagnosis codes, categorizing them into similar sections based on their respective diagnosis chapters [28,29].

We grouped all diagnosis codes according to ICD 9/10 classifications, resulting in 15 overarching categories of general pre-existing conditions: Circulatory, Digestive, Emergency, Endocrine, Eyes and ENT, Genitourinary, Infectious and Parasitic, Injury Poisoning, Mental and Behavioral Disorders, Musculoskeletal, Neoplasms, Nervous, Pregnancy and Childbirth, Respiratory, and Skin. For our study’s purposes, any patient with at least one diagnosis code falling under a general pre-existing condition category was classified as having that condition. This methodology allowed for a comprehensive and systematic analysis of pre-existing conditions among COVID-19 patients, enhancing the accuracy and relevance of our findings.

### Outcome

We use all-cause mortality as our primary binary outcome. All dead individuals in the OPTUM EHR data are marked with their death record of year and month. Only cases whose time of death follows the COVID-19 test order date are considered for our analysis.

### Statistical analysis

After data processing, we first conducted a two-sample unpaired t-test to find any differences in patients’ age between the alive and died populations. Secondly, we implemented a Chi-square test of independence between the mortality and the demographic variables such as age, gender, and race to determine the eligibility as predictors for our analysis. After verifying demographic variables as essential predictors, we then used multivariate logistic regression models adjusted for age, gender, race, ethnicity, and region to obtain pre-existing diagnosis codes that are influential to COVID-19 patients’ mortality.

To gain a more nuanced understanding, we implemented both joint and marginal analyses. In the joint analysis, we included all 117 pre-existing diagnosis codes in a single multivariate logistic regression model alongside the demographic variables to assess their combined effect on mortality. In contrast, the marginal analysis involved running separate multivariate logistic regressions for each of the 117 diagnosis codes, each adjusted for the same set of demographic variables, to evaluate the individual effect of each diagnosis code on the outcome. We then compared the results from these two approaches by calculating the Spearman’s correlation coefficient for the z-values obtained from each analysis. Additionally, we computed the Spearman’s correlation coefficient for the adjusted odds ratios to further compare the joint and marginal models’ outcomes.

Furthermore, we extended this approach to the 15 general pre-existing conditions, applying both joint and marginal logistic regression models adjusted for demographic variables. This comprehensive examination allowed us to assess not only the influence of specific diagnosis codes but also general categories of pre-existing conditions on COVID-19 mortality, providing a more comprehensive understanding of the factors contributing to patient outcomes.

## Results

### Demographical factors impacting COVID-19 deaths in the U.S

Table 1 presents an in-depth examination of demographic factors associated with the life status of COVID-19 patients at the end of our study period. The first row of the table displays the age, as in years, versus life status, a binary outcome of COVID-19 patients at the study’s conclusion. A notable observation from the table is that older individuals are more susceptible to being at risk of death than others. The median age for patients who survived is 46 with an interquartile range of 32 while the median age for patients who died is 78 years with an interquartile range of 18. We obtained a mean of 45.44 years for the alive group and a mean of 75.51 years for the deceased group. A two-sample t-test was performed on age based on life status. The null hypothesis for this t-test is that both means of the alive and deceased populations are equal. We reject the null hypothesis at a significant level of 0.05 and conclude that the population means are different (p-value <  0.001).

**Table 1:** Demographic characteristics based on life status.

Total (N = 223,286)		Alive (N = 218,831)	Deceased (N = 4455)	P - Value
**Age (in years)**	**mean (SD)**	45.44 (23.7)	75.51 (13.3)	**<0.001**
**Gender**	Male	96,313 (44%)	2,531 (56%)	**<0.001**
	Female	122,518 (56%)	2,401 (44%)	
**Race**	Caucasian	189,205 (87%)	3,871 (87%)	**0.001**
	African American	25,062 (11%)	526 (12%)	
	Asian	4,564 (2%)	58 (1%)	
**Ethnicity**	Hispanic	10,193 (5%)	162 (4%)	**0.002**
	Not Hispanic	208,638 (95%)	4,293 (96%)	
**Region**	Northeast	465,27 (21%)	750 (17%)	**<0.001**
	Midwest	131,676 (60%)	2,362 (53%)	
	South	34,919 (16%)	1,180 (26%)	
	West	5,709 (3%)	163 (4%)	

Additionally, Table 1 presents the detailed representation of other demographic variables counts along with p-values of the Chi-square test of independence. The EHR database provides a large body of evidence that COVID-19 death is impacted by gender, race, ethnicity, and region. All gender, race, ethnicity, and region hold p-values as listed in Table 1 which is less than 0.05 hence claiming that there are relationships between these demographic variables and the mortality. This supports the validity of utilizing these demographics as predictors for a logistic regression to remove their effect.

A crucial aspect of gender characteristics is displayed in Table 1. Female patients identified as alive are observed as the majority over male patients by 56%. On the other hand, COVID-19 patients identified as male and have died hold the majority of the deceased population by 56%. Caucasians hold the majority over other races in both alive and died population by 87% and 87% respectively. Likewise, Not Hispanic hold both the majority in alive and died populations by 95% and 96% respectively. In addition, Midwest holds the 60% in the alive population and 53% in the died population making it the region where most of our COVID-19 patients originated. The second biggest region for the alive population is the Northeast holding 21%, however the South region is behind the Midwest by 26% for the died population.

### Demographics-adjusted pre-existing diagnosis codes associated with the COVID-19 mortality in the U.S

Table 2 displays the odds ratios of the demographic and the 29 significant pre-existing diagnosis codes that are found to be associated with the COVID-19 mortality out of the 117 diagnosis codes with the joint logistic regression model fit (p-value <  0.05). Our data provides strong statistical evidence that 13 of the distinct 15 general categories were found to be a significant category that influences the risk to COVID-19 mortality. Among the demographics variables, age, gender, race, and region are found to be statistically significant.

**Table 2:** Adjusted Odds Ratio and P-value results of the joint logistic regression.

Variable		OR	95% CI of OR	P-value
**Age**		1.09	(1.08-1.09)	**<0.001**
**Gender**	Male	Reference		
	Female	0.66	(0.61 - 0.72)	**<0.001**
**Race**	Caucasian	Reference		
	African American	1.19	(1.07 - 1.33)	**0.01**
	Asian	0.86	(0.64 - 1.14)	0.49
**Ethnicity**	Hispanic	Reference		
	Not Hispanic	1.2	(1.0 - 1.43)	0.14
**Region**	Northeast	Reference		
	Midwest	1.37	(1.13 - 1.66)	**<0.001**
	South	1.18	(1.09 - 1.28)	**<0.001**
	West	1.61	(1.49 - 1.74)	**0.01**
**Circulatory**	Chronic rheumatic heart disease	0.67	(0.48 - 0.92)	**0.05**
	Other forms of heart disease	1.18	(1.09 - 1.28)	**<0.001**
**Endocrine**	Diabetes mellitus	1.14	(1.03 - 1.26)	**0.041**
	Other metabolic and immunity disorders	0.82	(0.75 - 0.90)	**<0.001**
**Emergency**	Provisional assignment of new diseases of uncertain etiology or emergency use	1.61	(1.49 - 1.74)	**<0.001**
**Genitourinary**	Diseases of male genital organs	0.8	(0.73 - 0.88)	**<0.001**
	Other diseases of urinary system	1.12	(1.03 - 1.21)	**0.032**
	Renal failure	1.76	(1.61 - 1.93)	**<0.001**
	Urolithiasis	0.82	(0.70 - 0.95)	**0.041**
	Nephritis, nephrotic syndrome, and nephrosis	0.86	(0.78 - 0.95)	**0.014**
**Mental and Behavioral Disorders**	Mood [affective] disorders	1.24	(1.12 - 1.36)	**<0.001**
**Nervous**	Cerebral palsy and other paralytic syndromes	0.84	(0.76 - 0.94)	**0.008**
**Respiratory**	Acute upper respiratory infections	0.78	(0.71 - 0.86)	**<0.001**
	Acute respiratory infections	0.87	(0.79 - 0.97)	**0.047**
	Other acute lower respiratory infections	0.83	(0.74 - 0.93)	**0.008**
	Other diseases of the respiratory system	2.0	(1.84 - 2.18)	**<0.001**
	Other diseases of the upper respiratory tract	0.87	(0.8 - 0.96)	**0.018**
	Chronic obstructive pulmonary disease and allied conditions	1.18	(1.06 - 1.32)	**0.018**
	Influenza and Pneumonia	1.53	(1.41 - 1.67)	**0.017**
**Neoplasms**	Benign neoplasms	0.79	(0.71 - 0.89)	**<0.001**
	Coagulation defects, purpura and other hemorrhagic conditions	1.37	(1.22 - 1.54)	**<0.001**
	Aplastic and other anemias	1.22	(1.12 - 1.34)	**<0.001**
**Musculoskeletal**	Other joint disorders	0.85	(0.77 - 0.92)	**0.001**
	Other dorsopathies	0.84	(0.76- 0.92)	**0.002**
	Arthrosis	0.88	(0.81 - 0.96)	**0.02**
**Skin**	Infections of the skin and subcutaneous tissue	1.15	(1.04 - 1.27)	**0.011**
**Infectious and Parasitic**	Other bacterial diseases	1.45	(1.31 - 1.61)	**<0.001**
**Injury Poisoning**	Injuries to the head	1.27	(1.1 - 1.46)	**0.007**
**Pregnancy and Childbirth**	Complications mainly related to pregnancy	2.95	(1.4 - 6.23)	**0.020**

Among 13 significant general pre-existing diagnosis categories, 11 categories have at least one diagnosis code with an odds ratio greater than 1. The most significant one being the respiratory category, specifically Other diseases of the respiratory system diagnosis, such as acute chest syndrome and chronic respiratory failure (for full details refer to ICD 10 codes J95-J99 and ICD 9 codes 510 -519), has 100.2% increase in the odds of COVID-19 mortality and hold the highest adjusted odds ratio compared to other respiratory diseases. Interestingly, renal failure is identified to pose the second highest increase in the likelihood of COVID-19 mortality. In contrast, seven of the 15 general categories have at least one diagnosis code that possesses an odds ratio less than 1. All diagnoses identified significant under musculoskeletal disorders are observed to decrease the odds of COVID-19 mortality. In addition, other metabolic and immunity disorders, such as cystinosis and obesity (refer to ICD 9 codes 270-279), identified in the endocrine category, hold a decrease in COVID-19 mortality odds.

### Statistical significance supported by the agreement between marginal and joint analyses

We implemented two logistic regression strategies: a joint model accounting for all demographic factors and diagnosis codes and a marginal model assessing each of the 117 diagnosis codes individually with adjustments for demographic variables. The joint model mitigates confounding effects but is prone to multicollinearity, potentially skewing results. However, the marginal model acts as an essential counterpart, allowing the comparison of adjusted odds ratios to gauge the influence of individual diagnosis codes. The similarity between corresponding adjusted odds ratios from the two models bolsters confidence in the significance of specific diagnosis codes, suggesting that their effects are robust to the presence of other variables.

**Fig 1:**
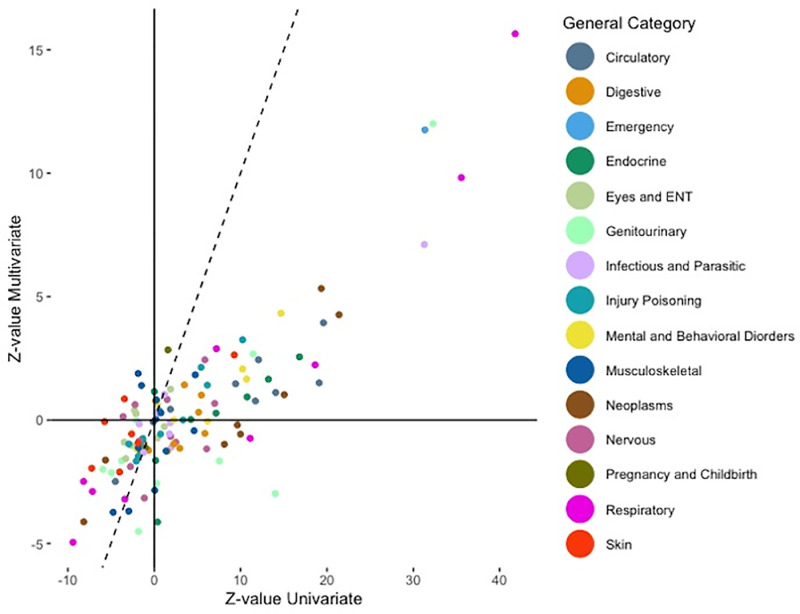
Scatterplot of Z-values on diagnoses given by joint and marginal analyses.

**Fig 2:**
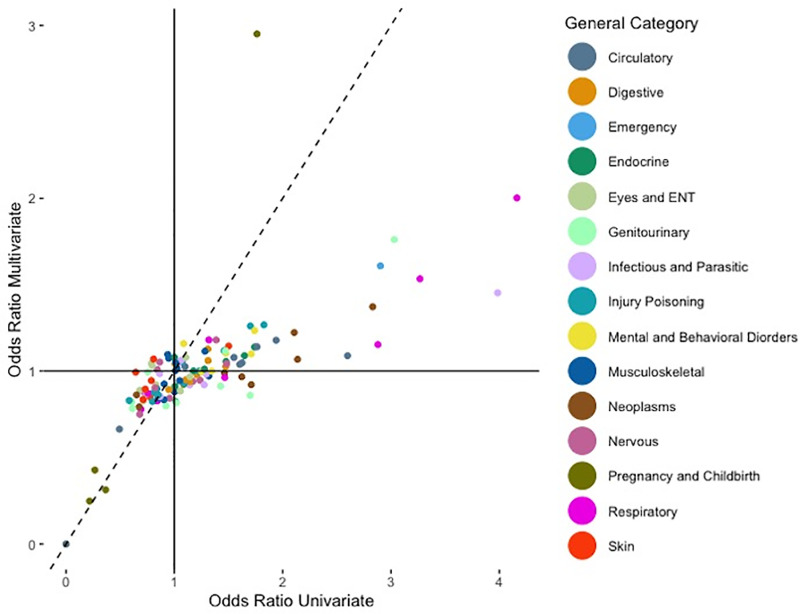
Scatterplot of adjusted odds ratios on diagnoses given by joint and marginal analyses.

The diagnosis z-values from the joint logistic model are plotted against the marginal diagnosis’ z-values in Fig 1. Similarly, the scatterplot represented in Fig 2 presents the associations between the adjusted odds ratios of the pre-existing diagnosis codes from the joint logistic model and the marginal logistic models. We obtained a spearman correlation coefficient for the z-value of 0.6818, which signifies that there is positive agreement between our main joint logistic model’s z-values and each individual marginal model’s z-values. We found a similar conclusion on the adjusted odds ratio scatterplot wherein the spearman coefficient for the adjusted odds ratio is 0.7651.

**Fig 3:**
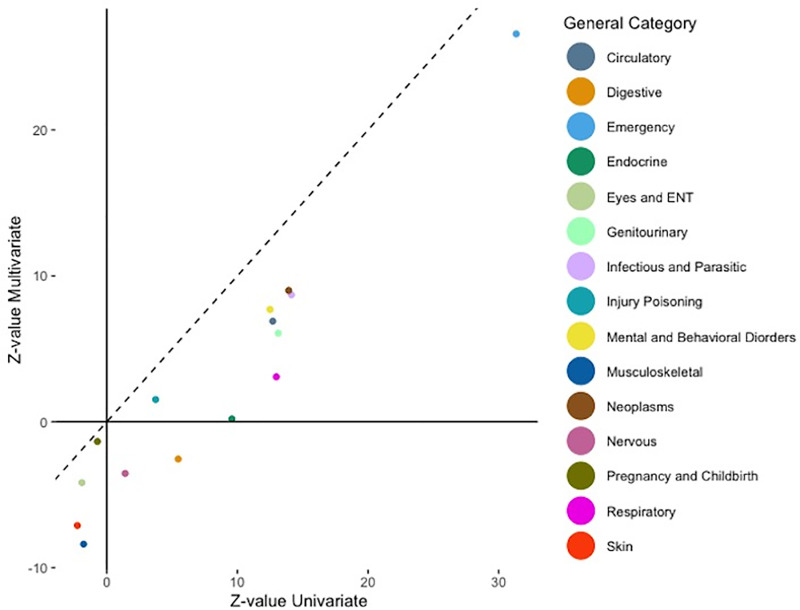
Scatterplot of Z-values on general categories given by joint and marginal analyses.

**Fig 4:**
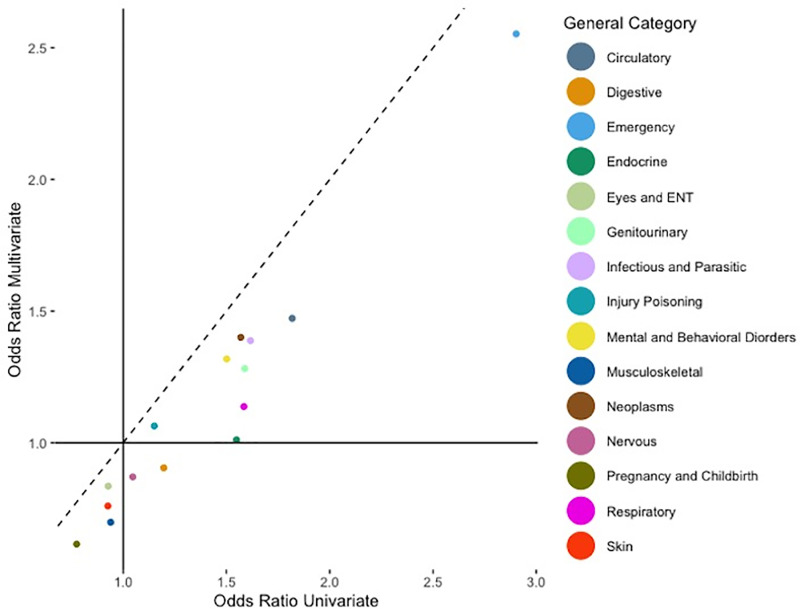
Scatterplot of adjusted odds ratios on general categories given by joint and marginal analyses.

We found a great agreement on the z-values from joint analysis and marginal analyses from the scatterplots of the overall general pre-existing diagnosis categories in Fig 3. Similarly, in Fig 4 the adjusted odds ratios are well agreed. The Spearman correlation coefficients further substantiate this alignment, with both the general z-values and the general adjusted odds ratios showing a strong correlation of 0.93.

Overall, the pre-existing diagnosis codes identified by the joint analysis with the demographic adjustment are substantiated by the results from the marginal analysis. The results from the joint logistic model with the 15 general categories are also supported by the marginal analysis; therefore, our conclusion is that the 13 general diagnosis categories pose significant association with the COVID-19 mortality.

## Discussion

Among the COVID-19 positive patients spanned across the United States until January 2021, our analysis highlighted many patient characteristics and underlying medical diagnosis codes to be associated with COVID-19 mortality. The logistic model displayed that all the demographic variables excluding ethnicity have prominent influence in determining COVID-19 mortality. The sample of COVID-19 positive cases obtained from the comprehensive OPTUM networks (*N =  223,286*) is large enough to generalize our study results to COVID-19 patients in the U.S population. Patients who are identified with the following characteristics: Male African American from the west region posed a greater risk in observing COVID-19 mortality. COVID-19 patients from the northeast region of the United States have lower odds in observing COVID-19 mortality when compared to the west region holding the highest odds in experiencing mortality.

A history of respiratory diseases, circulatory disease and diabetes hold higher odds in experiencing COVID-19 death. Such pre-existing diseases were reported as a predictor of COVID-19 mortality in the literature [4,30–35]. In our current model, we have found nine new diagnoses such as Renal Failure, and Other diseases of urinary system, which are shown to hold higher odds in observing COVID-19 death. Among these diagnoses, Renal Failure presents a complex challenge in terms of determining whether the primary disease process leading to this association is chronic kidney disease (CKD), acute kidney injury (AKI), or AKI on CKD, further complicated by the necessity of dialysis [36]. This intricate relationship underscores the need for precise diagnostic differentiation to understand the exact nature of the association.

A COVID-19 study [26] observed that people residing in the Northeastern region of the United States have the highest risk of death, however we have found that the COVID-19 patients identified from the West region (OR: 1.61, p-value: 0.01) are more susceptible to observe COVID-19 mortality than the Northeast [26]. Note that our results based on the COVID-19 cases examine the fatality of the disease among the infected patients while [26] explored the risk of COVID-19 related death of the entire population. Additionally, in the findings of [25] a lower COVID-19 mortality for African American patients was detected among hospitalized patients, while our results show that African American patients hold (OR: 1.19, p-value: 0.01) an increased risk of COVID-19 mortality. Such a difference on the risk of death may be attributable to limited access to hospitalization for African American patients [37]. Further interrogations are needed.

Characteristics of COVID-19 patients admitted to hospitals were carefully investigated in [21,38,39], each of which used OPTUM, Geisinger, and three U.S. EHR databases (Academic Health System, Explorys, and OneFlorida), respectively. The results in [21,39] are based on descriptive statistics of variables characterizing hospitalized patients, not establishing statistical significance on their findings. [38] investigated patients’ phenotypes that determine their hospitalization status with Firth’s logistic regression [40] adjusted for age, sex and race, which is similar to our marginal analysis. [41] studied COVID-19 mortality with 31,461 patients from TriNetX EHR data using multivariate logistic regression. Our study results that are similar to those in [41] have statistical power increased due to a much larger dataset of 223,286 COVID-19 patients, together with our validation through the marginal analysis.

In the analysis with the 115 pre-existing diagnostic codes, several diagnostic codes including chronic rheumatic heart disease and other metabolic and immunity disorders hold lower odds in experiencing death by COVID-19, which is difficult to find supporting evidence from previous studies. Although considering the pre-existing diagnostic codes altogether is useful for evaluating specific pre-existing diagnoses, high correlations among the diagnostic codes may underestimate or overestimate the odds, misleading the results. The analyses with the general pre-existing conditions mitigate such high correlations to some extent, by merging pre-existing diagnoses under a general category. From the general analyses, all general pre-existing conditions but Digestive and Eyes and ENT are found to be significant for explaining COVID-19 mortality. We find that Circulatory and Endocrine disorders that include a diagnosis code that possesses an odds ratio less than 1 are found to hold higher odds in the COVID-19 mortality.

This study faced some limitations. There is less than a 1 percent rate of uninsured individuals in the EHR database, which is far less than that in the U.S. Thus, poverty might confound the effect of the demographic characteristics on the COVID-19 mortality. Next, use of all-cause mortality may overestimate the COVID-19 mortality. Obtaining information about causes of death would reduce such bias. Another limitation comes from the design of the dataset, which does not record the death date and the cause of the patients’ death. Such information would have allowed researchers to conduct survival analysis and various other time series analysis that could have enhanced our knowledge on which diagnoses posed a significant influence on the cause of COVID-19 mortality. The current study does not delve into comorbidity due to the limitation of the logistic model’s complexity. Further study of comorbidity on this large dataset would produce vital information into learning the interconnection between diagnoses and COVID-19 mortality [10].

Our data curation strategy for this study primarily revolved around the inclusion of all COVID-positive patients with complete demographic information. Subsequently, we collected their pre-existing diagnoses and selected only the top five hundred diagnosis codes. Consequently, certain diagnoses associated with specific diseases might not have been fully represented in the dataset. For instance, while rheumatic disease is linked to a higher risk of COVID-19 mortality, multiple diagnosis codes pertaining to this disease may not have been captured [42]. As a result, individual diagnosis codes such as ‘other joint disorders’, ‘other dorsopathies’, and ‘arthrosis’ exhibit lower odds of COVID-19 mortality. Another issue of our marginal analysis is that we did not control for other pre-existing diagnosis codes when evaluating a given individual diagnosis code. This might hinder or bias the association between that diagnosis code and the COVID-19 mortality. Controlling for other pre-existing diagnosis codes could provide additional insight into such association [22].

Additionally, future exploration of the determination of COVID-19 mortality in various clinical settings such as hospitalization, ICU, urgent care, and others currently identified in OPTUM data would help advance our healthcare practices and operations. Furthermore, determining the age of COVID-19 patients who are alive is an obscure process since due to the substantial size of visit files and our current computing memory and processing speed, obtaining the most recent visit date for each patient is an impractical task. Obtaining the accurate current age of living COVID-19 patients would deepen our knowledge on understanding the association to COVID-19 mortality based on age groups.

## Conclusion

Recognizing patient health diagnosis and characteristics with COVID-19 death is vital for aiding public awareness and asserting better precautionary measures. Through 223,286 patient EHR, we were able to conduct a large cohort logistic analysis. With respect to pre-existing diagnoses, patients who have had one of the following: complications mainly related to pregnancy, other diseases of the respiratory system, renal failure, influenza and pneumonia, other bacterial diseases, coagulation defects, purpura and other hemorrhagic conditions, injuries to the head, mood [affective] disorders, aplastic and other anemias, chronic obstructive pulmonary disease and allied conditions, other forms of heart disease, diabetes mellitus, and other diseases of the urinary system are deemed to pose a greater odds ratio in exhibiting COVID-19 mortality. Our large cohort analysis can pave the way for future healthcare policies and outbreak preparedness plans.

## Supporting information

S1 Fig Steps in selection of COVID-19 patients.
(TIF)
