# Supplementary figures and images for: Health diagnosis associated with COVID-19 death in the United States: A retrospective cohort study using electronic health records
Source: PLoS One. 2025 Mar 31;20(3):e0319585. doi: 10.1371/journal.pone.0319585 (PMC11957315; doi:10.1371/journal.pone.0319585)

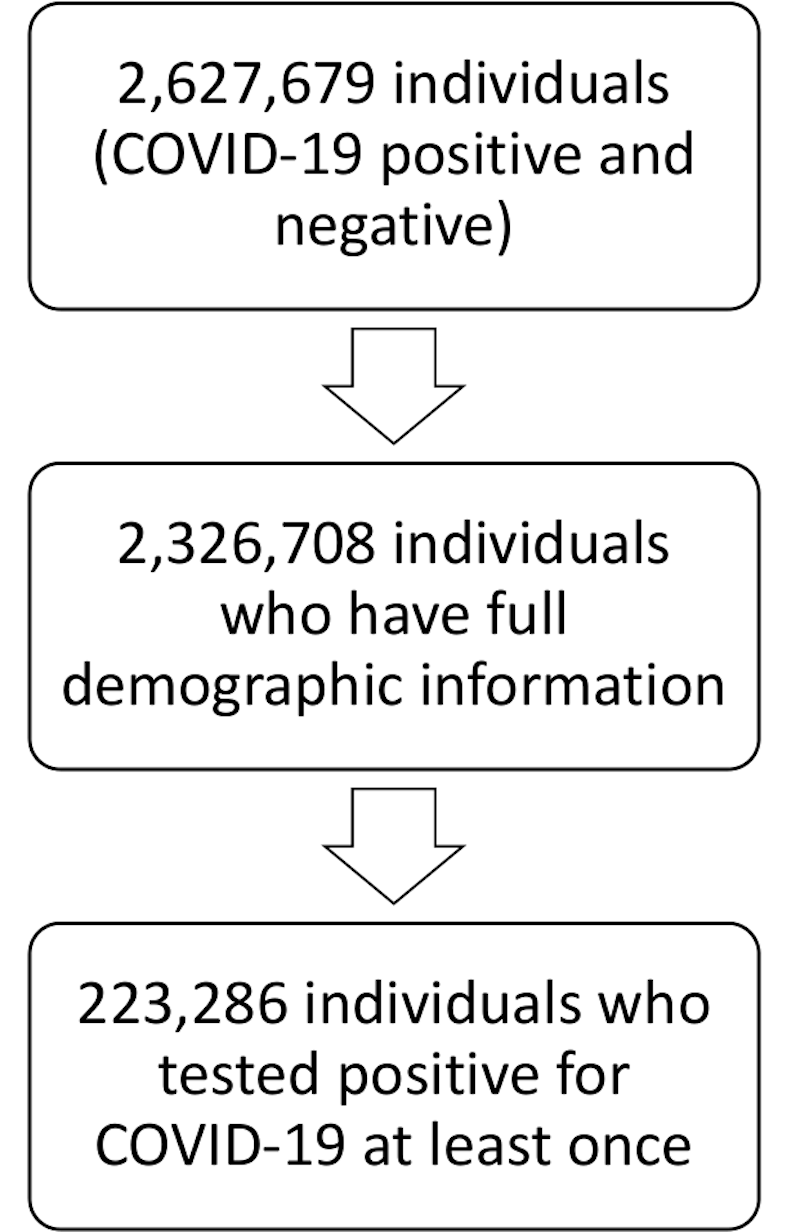

Supplement: S1 Fig — (TIF) [file pone.0319585.s001.tif]
